# Supplementary material for: RIPK3 inhibitor GSK872 targets angiotensin II induced cardiomyocyte hypertrophy by regulating Ca2+/ calmodulin dependent protein kinase II
Source: PLoS One. 2026 Jan 7;21(1):e0334387. doi: 10.1371/journal.pone.0334387 (PMC12779033; doi:10.1371/journal.pone.0334387)
Supplement: S1 File — (PDF) [file pone.0334387.s001.pdf]

Minimal data

Figure 1.

ANP

Control	AngII
0.8597673	1.907130
0.984374	1.591043
1.245664	1.689360
1.055181	1.639786
0.9358118	1.487170
0.9192023	1.421081

BMP

Control	AngII
0.8597673	2.318729
0.984374	1.765043
1.245664	1.675936
1.055181	1.968594
0.9358118	1.677170
0.9192023	1.675481

RIPK3

Control	AngII

1.016038	2.094755
1.232047	2.131377
0.8474923	2.303259
0.9771252	2.721535
0.9970242	1.963218
0.9302731	1.858941

p-RIPK3

Control	AngII
0.8773364	2.120905
0.9105206	2.032163
0.9993311	2.398027
1.381981	2.119914
0.8333692	2.030401
0.9974617	1.610658

Figure 2.

ox-CAMKII

Control	AngII
1.452073	1.808796
0.9278637	1.334703
0.8796923	1.472006
0.8730597	2.019845

0.9309416 1.778916

0.9363695 2.306823

p-CAMKII

Control AngII

1.035521 2.123844

0.7234917 2.068946

1.380335 2.487013

1.035521 2.123844

0.7234917 2.068946

1.380335 2.487013

CaMKIIδ

CaMKIIδA	1.129200	1.005220	0.7767157	0.9407527	1.182528	0.9655843	0.6327716	0.6233177	0.9244923	0.7682246	0.4826981	0.4976824
CaMKIIδB	1.036408	1.178397	0.8950084	0.9339924	0.9338886	1.022306	0.7610782	0.4949614	0.7714691	0.7297029	0.5849968	0.4392422
CaMKIIIδC	1.176787	0.9654111	0.9708562	0.9571413	0.9524078	0.9773968	1.603001	1.264272	1.534870	1.769258	1.344585	1.523829

Figure 3.

Tunnel

Control AngII

0.8864207 3.106727

1.370224 3.893423

0.8405386 3.134118

0.8199926 3.146193

1.166708 3.243234

0.9161156 2.951371

Cleaved-caspase3/ caspase3

Control	AngII
0.8023481	2.069046
1.275350	1.834257
0.7751506	1.829811
0.7996699	1.966782
0.8135635	3.524361
1.533918	2.367121

LDH

Control	AngII
0.6221861	3.681817
0.923406	3.237930
1.205440	3.426311
0.8302976	5.965178
1.390386	3.124176
1.118795	3.725177
0.7888247	3.770125
1.120664	3.861907

ATP

Control AngII

0.9212862	0.3900095
0.9303781	0.382473
0.9267415	0.3900086
0.929266	0.3310897
0.8789333	0.3010271
1.746747	0.3857611
0.666648	0.3151355

Figure 4.

ANP

control	AngII	GSK'872+ Ang II	GSK'872
1.069823	1.668051	0.7794057	0.884627
0.6141283	1.554697	0.780037	0.9331796
1.122835	1.867782	0.835331	0.9555772
1.047751	1.708778	0.997324	0.8723712
1.082941	1.921364	0.8851667	0.7823468
1.062522	1.805922	0.8117822	0.8375006

BNP

Control	AngII	GSK'872+Ang II	GSK'872
0.8597673	2.382762	1.378672	1.218672
0.984374	2.933282	1.562763	1.318792

1.245664	1.982168	1.776268	1.278973
1.055181	1.987637	1.728183	1.372817
0.9358118	1.768120	1.671820	1.127612
0.9192023	1.997298	1.621764	1.027879

RIPK3

control	AngII	GSK'872+Ang II	GSK'872
0.8306352	1.463588	1.750029	0.5764064
1.564459	2.624677	1.168503	0.5235476
0.9720651	1.703377	1.285052	0.4604192
0.8400779	1.775925	1.142627	0.457956
0.9812015	1.885534	1.252279	0.4241821
0.8115616	1.723043	1.156698	0.2245449

p-RIPK3

control	AngII	GSK'872+Ang II	GSK'872
0.9764161	1.583720	0.3476327	0.227832
1.257774	1.681443	0.337826	0.2237687
0.7927235	1.594248	0.323572	0.3675367
1.036449	1.609468	0.325372	0.325371
0.8660704	1.746338	0.2328687	0.3675638
1.070568	1.539126	0.338682	0.371573

Figure 5.

ox-CAMKII

control	AngII	GSK'872+Ang II	GSK'872
1.259466	1.433703	0.9467852	0.9691366
0.940983	1.658592	0.9126943	0.990085
0.9232318	1.630338	0.7218932	0.6249736
0.9364983	1.738126	0.8055058	0.7310067
0.997901	1.878899	0.8613235	0.8475342
0.9419201	1.712725	0.840988	0.8913763

p-CAMKII

control	AngII	GSK'872+Ang II	GSK'872
0.8938425	2.015102	1.130527	0.9017029
1.214440	2.441927	0.9733128	1.127275
1.024801	2.426354	0.9016374	0.9788815
0.9371435	2.339605	1.015216	1.188317
0.9218239	2.337592	0.9676864	0.9614317
1.007949	2.425907	0.9928461	0.9224332

Figure 6.

ASF

control	AngII	GSK'872+Ang II	GSK'872

1.104773	1.904444	1.237843	1.191081
0.9088051	2.268936	1.263848	0.9304685
0.8980352	1.963783	1.281744	0.9606684
0.9809582	1.823794	1.293787	0.8214215
1.135388	1.938970	1.398478	0.9443259
0.9720408	1.738924	1.299379	0.968881

SC35

control	AngII	GSK'872+Ang II	GSK'872
1.120899	2.067122	1.381721	1.201218
0.8067983	2.127671	1.314171	1.181058
0.969292	2.002794	1.230785	1.178878
0.9782703	2.108279	1.120664	1.196085
1.050264	2.072871	1.251731	1.350547
1.074476	1.978162	1.158214	1.309196

CaMKIδ

CaMKIIδA	1.142131	1.007261	0.962718	0.967286	0.928121	0.982871
CaMKIIδB	1.082712	1.017627	0.9862812	0.992817	0.916257	1.089710
CaMKIIIδC	1.061257	0.912790	1.019279	1.127811	0.917852	1.081756
0.467120	0.5617562	0.612587	0.526710	0.629187	0.526781	
0.516762	0.498390	0.538712	0.617868	0.738678	0.4623523	
1.782812	1.438973	1.729786	1.669258	1.476212	1.518273	
0.821767	0.821973	1.317823	0.897270	0.7187892	0.819721	
0.981687	0.971820	0.718627	0.826782	0.886870	0.8819793	
1.137829	1.128939	1.138712	1.017826	1.238711	1.318792	
1.019828	1.012873	0.9178277	0.9183123	1.017263	0.929793	
1.092764	1.182779	0.917831	0.9167573	0.9917564	1.075370	
1.127860	0.9927867	0.983612	0.991786	0.917283	0.997868	

Figure 7.

Tunnel

control	AngII	GSK'872+Ang II	GSK'872
0.9283224	3.279839	2.436226	0.8021967
1.109015	4.491649	2.080314	1.081697
1.098504	3.616393	2.267745	0.913597
0.9183881	3.875380	2.214956	0.8908272
0.976762	3.245650	1.698745	0.9953687
0.9690081	3.470239	1.611370	0.7103916

Cleaved-caspase3/ caspase3

control	AngII	GSK'872+Ang II	GSK'872
0.9820257	2.017055	1.525215	1.061220
1.039128	2.525591	1.790713	0.9514356
0.8313315	1.965176	1.302752	1.358867
0.9534109	1.846162	1.338097	1.253012
1.080929	2.125379	1.371473	1.050938
1.113175	2.321335	1.470209	1.073768

Figure 8.

MDA

control	AngII	GSK'872+Ang II	GSK'872
2.021213	4.623720	2.267730	1.372830
1.576423	4.874832	2.973670	2.162783

1.927630	4.746733	2.265360	2.524530
2.038971	4.276312	2.953670	1.867313
1.987276	4.365320	2.926732	1.961731
1.865360	4.265340	2.232310	1.938687

T-AOC

control	AngII	GSK'872+Ang II	GSK'872
85.	60.	77.	80.
80.	55.	78.	78.
82.	54.	70.	80.
89.	62.	75.	79.
78.	61.	70.	81.
70.	66.	66.	77.

SOD

control	AngII	GSK'872+Ang II	GSK'872
130.	90.	180.	177.
155.	82.	150.	156.
134.	100.	158.	179.
132.	105.	160.	166.
139.	110.	163.	176.
132.	112.	159.	170.

Figure 9.

LDH

control	AngII	GSK'872+Ang II	GSK'872
1.179077	2.622659	1.575605	1.012791
1.023755	3.309731	1.442211	1.188214
1.069438	3.529009	2.173138	0.9305619
0.7715852	2.359525	1.827775	1.126085
1.356327	4.137506	1.469621	1.294198
0.6034719	3.061215	1.860667	1.113294
1.093193	2.900411	2.116492	0.7734125
0.9031521	2.714025	1.736409	1.131567

ATP

control	AngII	GSK'872+Ang II	GSK'872
0.7967802	0.4900776	0.7658985	0.9523059
1.059993	0.4987756	0.7759529	0.9636372
1.037610	0.4977382	0.8209988	1.031545
1.046986	0.4353364	0.7693697	0.9331943
1.057599	0.5237523	0.7587566	0.9643553
1.001421	0.4780681	0.740044	0.8275821
1.040522	0.4446728	0.818565	0.9476377

0.9590887 0.4598343 0.781060 0.9318777
